# “Getting Used to It, but Still Unwelcome”: A Grounded Theory Study of Physical Identity Development in Later Life

**DOI:** 10.3390/ijerph18189557

**Published:** 2021-09-10

**Authors:** Bora Jin, Elizabeth A. Roumell

**Affiliations:** 1 The Gerontology Institute, Georgia State University, Atlanta, GA 30303, USA; 2 Department of Educational Administration & Human Resource Development, Texas A&M University, College Station, TX 77843, USA; earoumell@tamu.edu

**Keywords:** perception of aging, physical activity, physical change, identity process, coping strategies

## Abstract

Given the global trends toward an aging society and the increased desire for healthy aging in late life, this study examines older adults’ perceptions of aging and their physical identity through their engagement in physical activities. Adopting a grounded theory, we interviewed 15 individuals aged 65 years and older, who were involved in physical activities on a regular basis. This study provided a final model depicting (a) divergent and convergent modes of strategies and socioemotional aspects of physical identity development in later life and (b) different strategies employed between younger-old versus older-old age groups and between participants who have underlying health conditions and those who do not. These findings add a contextual explanation of identity development in later life and stress the recurring process of physical identity development.

## 1. Introduction

Most people in later life go through major life events, including retirement, children leaving home, loss of a near one, the onset of physical illness or injury, and changes in physical ability; thus, they experience signals that the end stage of life is approaching [1,2]. Such life-turning events often provide older adults with an opportunity to contemplate themselves and reflect on how and why they have made a right or a new decision to cope with the changes [3]. In this way, undergoing physical changes and critical life events and recognizing the limitation of time in their lives may catalyze older adults to develop new attitudes and personal motivation to become more involved in meaningful activities [4].

Some of the most influential life circumstances that older adults grapple with are health-related issues [1]. Particularly, maintaining physical activity is a critical element of older adults’ health, physical functioning, and overall well-being [5]. In line with the older adults’ desire to remain physically and mentally active without illness [6,7], regular physical activity for older adults needs to be emphasized because it contributes to the benefits of improving fitness (e.g., balance, cardiovascular function, and muscle and bone mass), preserving physical function (e.g., mobility, fewer falls, and independent living) [8,9], and supporting positive mental health and healthy aging [10].

When engaging in physical activity, older adults may experience a transformation of “feelings of control and autonomy and the development of an identity as an exerciser” [11] (p. 186). In this metacognitive process of building physical self-concept, older adults develop a higher degree of commitment to behaviors that correspond with their desired physical sense of self [12]. A sense of self in the physical domain, such as perceptions of competence related to physical tasks or appearance of one’s body, is an integral part of the self-concept [13,14]. Physical identity can be a large part of one’s overall identity that connects to other relevant identities in their larger self and social-related contexts [15,16]. Physical identity is directly tied to an older adult’s personal sense of autonomy, independence, not wanting to be a burden on others, desire to make meaningful contributions and to stay relevant, desire to be there for others as long as possible, and a sense of pursuing his/her life not yet lived while there is still time [17,18,19].

Extant studies on physical identity among older adults have examined relationships or associations between older individuals’ physical identity and their physical activity or health behaviors. For instance, Strachan et al. [20] showed that a stronger physically active identity was associated with higher self-regulatory efficacy, more frequent physical activity, and greater life satisfaction. Pelssers et al. [21] revealed that older individuals who internalize a stronger physical identity were also more likely to be autonomously motivated to exercise. While there are a few studies focusing on engagement in physical activities and identity among older adults [22,23], few scholars have investigated the way or process in which older adults construct or maintain their physical identity; we located ourselves in one of the very few studies on theorizing the process of identity development through engagement in physical activity among older adults. In this study, we examined older adults’ perceptions of aging and their physical identity through their engagement in physical activities. The following research questions guided the study: (a) How does older adults’ participation in particular physical activities affect their perceptions of aging? (b) How does older adults’ engagement in physical activity shape their identity in later life? 

## 2. Methods

We adopted Strauss and Corbin’s [24,25] version of grounded theory to develop a substantive theory accounting for the specific factors and their relationships comprising the process of older adults’ physical identity development. For the final theoretical conceptualization, a theoretical diagram aided us in visualizing various categories and presenting a final theory.

### 2.1. Data Collection

From February to July 2020, we recruited individuals aged 65 years and older who were involved in physical activities on a regular basis. Purposeful sampling allowed us to select individuals and sites for this study, such as community recreation or wellness centers, community-based senior centers, parks, golf courses, and sports clubs in the local area. All data were collected from the individuals residing in independent living in an urban area in the South Central state. Before reaching out to the research sites and the potential participants, we obtained approval for this study by the appropriate Institutional Review Board (IRB). In order to do that, a recruitment letter, a recruitment flyer, a letter of support from the relevant facilities, and a participant consent form were collected and submitted to the IRB. The means to collect the data was individual interviews. An interview is a particular kind of conversation in which knowledge is constructed in the interaction between the interviewer and the interviewee that requires active asking and listening [26]. Prior to the interview, we created key questions; then experts who specialize in adult education, educational gerontology, and health and kinesiology evaluated the interview guide. During the interview, we allowed for flexibility to ask additional questions to discover new ideas and categories [27]. Each interview lasted approximately 60 min. We used the software (Otter ai^®^, Mountain View, CA, USA) for the initial transcripts, listened again to each interview, and proofread it. All recorded data were coded by using pseudonyms and de-identified. Following the theoretical sampling method, we collected and analyzed data simultaneously that informed us to decide what data needed to be collected next for illuminating the emergent theory [28,29]. We conducted a few subsequent rounds of interviews until the data were fully saturated. In the initial round of data collection, seven face-to-face individual interviews and five phone interviews were carried out. In the second round of data collection, nine out of 12 participants from the first round responded to the interviewee’s phone call and were interviewed again on the phone. Then three more face-to-face interviews with face masks were added to the data analysis based on theoretical sampling.

### 2.2. Theoretical Sampling

Theoretical sampling, which is an important feature in grounded theory, refers to the iterative process based on categories that emerged from ongoing data analysis for generating theory [29]. To sample theoretically, we selected participants based on the descriptive needs of emerging concepts and categories [30,31]. We conducted our data analysis immediately after the first data were collected, so that information from the first rounds of data collection informed us what data needed to be collected next and where these data could be found ultimately to develop the emergent theory [28].

During the early rounds of data collection and constant comparative analysis, we identified different patterns between the younger-old group (60s–70s) and the older-old group (80s). However, more information was needed to find out about conditions that may interact with the categories under two age subgroups. While the younger-old group included both participants who had and had not had any underlying health conditions, the older-old group only included the participants with one to multiple underlying health conditions. Considering that age subgroups and underlying conditions may interact with the participants’ engagement in physical activity, we decided to collect more data that particularly fell into the category of the older-old group without underlying health conditions. Therefore, we conducted the third round of data collection to gather “the most information-rich source of data” [32] (p. 11) based on our analytical needs (see Table 1). Through theoretical sampling, we collected data from two more participants who were in their eighties, without underlying conditions. Additionally, data from one more participant who was in her seventies with an underlying condition were added by referral of one of the participants in the third round of data collection.

### 2.3. Data Analysis

Throughout the process of data collection and analysis, we used constant comparative analysis, using the software Atlas. ti Cloud^®^ (Berlin, Germany) to identify categories, aiming at the theory generation of older adults’ physical identity development process [24,25]. Conceptual terminology in grounded theory includes codes (i.e., important words or groups of words that symbolically assign the labels) and categories (i.e., groups of related codes) that are grounded in data [32]. The constant comparative method for data analysis helped us constantly compare “incident to incident, incident to codes, codes to codes, codes to categories, categories to categories”, and categories again back to raw data for generating highly abstract conceptual categories [32] (p. 11). In addition, the first author maintained her habit of writing memos throughout the data analysis including operational (i.e., the directions to the researcher–self, such as sampling and data collection), theoretical (i.e., the researcher’s inductive or deductive thinking about potentially relevant categories and their relationships), and code memos (i.e., conceptual labels, paradigm features, and indications of the process).

We carried out three phases of coding (i.e., open, axial, and selective coding). In the open coding, we carefully read the transcript and coded words and short phrases that struck us by using the direct language of the participants (in vivo coding) or a gerund form (i.e., *-ing* form of a verb). Particularly for the axial coding stage, we utilized an analytic tool of the paradigm model comprising phenomenon, causal conditions, context, intervening conditions, action/interactional strategies, and consequences [24]. This paradigm model led us to “think systematically about data and to relate them in very complex ways” [24] (p. 99).

In order to utilize the paradigm model, firstly, we started putting the categories from open coding under each element of the paradigm model. We placed the categories that prompted the participants’ initial desire for the phenomenon under the causal condition. For example, physical signs of aging, pain or distress from chronic disease, a higher probability of injury, and life-changing events were identified as determining the phenomenon of physical identity shift, along with the contextual conditions of personal trajectory in the physical domain, personal inclination for exercise or body image, affordability, and life responsibility. The categories that are a “particular set of conditions within which the action/interactional strategies are taken to manage, handle, carry out, and respond to a specific phenomenon” [24] (p. 101) were considered as a context. Next, we placed the categories that acted to either facilitate or constrain the strategies under the intervening condition. Categories, such as emotional distress, age-related stereotype, dutifulness, competence, empathy, support, driving mobility can either promote or inhibit adults’ strategies for managing physical identity shift. Action/interactional strategies included some categories for how the participants specifically respond to the phenomenon. We identified the categories, for instance, of determination, self-discipline, self-cognizance, reconciliation, moderation, and adaptation as signifying the process of a physical identity shift. The consequences of the actions and interactions included both the outcomes of the accomplishment of actions and/or the failure to take the action [24]. Satisfaction or ambivalence regarding their physical identity and transcendental views were considered to be consequences of the actions and interactional strategies to manage the phenomenon. Table 2 presents the first step of axial coding and categories under each element of the paradigm model.

Finally, selective coding refers to “the process of selecting the core category, systematically relating it to other categories, validating those relationships, and filling in categories that need further refinement and development” [24] (p. 116). In this advanced stage of coding, identification of the core category is a fundamental aspect that condenses the process apparent in the categories and subcategories [24,31]. In this regard, Strauss and Corbin [24] noted that a core category is “the central phenomenon around which all the other categories are integrated” (p. 116). Once a core category was identified, it propelled us to naturally progress to the advanced analysis by refining and integrating each theoretical component [32]. As a tool for theoretical conceptualization, a theoretical diagram aided us in visualizing various categories, systematically organizing all of the linking categories around the core category, finding the gaps in the advanced analysis, and thus ultimately illustrating a theoretical construction of the participants’ responses within the core category [24,33,34].

## 3. Results

### 3.1. Participants Overview

Fifteen individuals aged 65 and older who are physically active participated in the study. Active older adults were considered those who do at least 150–300 min of moderate-intensity physical activity a week or an equivalent amount (75–150 min) of vigorous-intensity activity; highly active older adults were considered those who do more than 300 min of moderate-intensity physical activity a week or an equivalent amount of vigorous-intensity activity (US Department of Health and Human Services, 2018) [9]. All participants’ levels of physical activity were active (60%) or highly active (40%). The sample included 73.3% females and 26.7% males. Participants’ age ranged from 67 to 89 years (60% were in their eighties, 26.7% were in their seventies, and 13.3% were in their sixties). The participants engaged in the physical activities of walking (*n* = 13), senior exercise programs (*n* = 9), gym (*n* = 4), self-workout at home (*n* = 5), golf (*n* = 5), water aerobics (*n* = 4), swimming (*n* = 1), and cycling (*n* = 1). In terms of the participants’ exercise status, 73.3% of them maintained an active lifestyle. Their exercise preferences were social exerciser (66.7%) or solitary exerciser (33.3%). Table 3 provides participants demographics. In the following sections, the categories are written in italics and the codes are written in underlined italics.

### 3.2. Core Category: Physical Identity Shift in Later Life

The core category refers to “the central phenomenon around which all the other categories are integrated” [24] (p. 116). The core category of this study is *physical identity shift in later life*. The core category was caused by *the physical signs of aging*, *pain or distress by chronic disease*, a *higher probability of injury*, and *life-changing events*. The participants began to notice the discrepancy between what they can and cannot do by the appearance of symptoms that they never worried about when they were younger. To deal with feelings of discrepancy derived from such new experiences, the participants pursued personal strategies in relationship with the influence of intervening conditions. It was evident that the participants went through divergent (i.e., the tendency to seek problem-solving by multiple ideas) [35,36,37] and convergent processes (i.e., the tendency to use existing multiple knowledge of life to analyze the given information or solve problems) [35,38,39] to fit their aging body or current physical and mental condition. Finally, physical identity shift resulted from these adjustments, where the participants reached the status of *satisfaction with their own physical identity, ambivalence regarding their physical identity*, or *transcendental views*.

### 3.3. Three Traits of Processes in Action/Interactional Strategies

Following Strauss and Corbin’s [24] paradigm model, particularly the categories of action/interactional strategy stood out as a primary process that supports how older adults develop or maintain their physical identity by engaging in physical activities that are personally satisfying as they age. Participants exhibited a divergent mode of coping strategies by actively seeking new challenges and anticipating that multiple solutions would effectively work for dealing with the issues. Participants also exhibited convergent strategies by knowing how to effectively resolve the issues in which their cumulative personal life experiences and what others have thought informed their decision. However, these two modes of the process were not seen as mutually exclusive but as sometimes operating simultaneously. Additionally, socio-emotional aspects (i.e., social interaction and retrospection) manifested in both divergent and convergent processes. The divergent process comprised seven categories, and the convergent process consisted of five categories. Table 4 provides three traits of the process in action/interactional strategies.

#### 3.3.1. Divergent Process

The divergent process was where the participants tended to be oriented toward individuality and seek problem-solving by exploring multiple ideas. The divergent process encompasses the following categories of (1) *determination*, (2) *self-discipline*, (3) *deliberation*, (4) *achievement striving*, (5) *active experimentation*, (6) *immersion*, and (7) *routinization*.

The first divergent phase begins with the participants’ dedicated decision to continue the activities they have been performing, despite the age-related physical or mental changes, and this is identified as *determination*. Eleven participants expressed a dedication to *not letting their body decline*. For example, Bonnie said, “I just don’t dwell on being older, I do the best I can with the exercises. So far so good”. When asked to describe the participants’ feelings about their body functioning, most of the participants acknowledged the physical changes but expressed their decisive attitude of *not allowing themselves to feel down*. Kayla said, “Sometimes I can’t do those leg lifts… But I’m not gonna let it frustrate me”.

The second phase in which the participants have the ability to begin the exercise-related tasks and carry them through to their goal achievement is identified as *self-discipline*. Thirteen participants showed self-disciplined attitudes in their exercise practice by *applying a rigorous standard to self*. One participant mentioned how he pushed himself to maintain a certain level of flexibility. Stanley stated, “I have to really force myself to remain flexible and be active… I’m critical of my body and I’m not overly, but I’m critical in my body because I say I lose the flexibility that upsets me, I need to be flexible”.

The third divergent phase, *deliberation*, was characterized as the participants’ careful consideration before taking action or situations that occur. Eight participants showed their attitude of being cautious about not pushing their physical limits too far. One participant mentioned that she was aware of her physical limitations or weak points when exercising and showed attentiveness. Sherry said, “I watch what I’m doing. And if I feel as though I’m going to get out of bounds, I don’t do that”. Some of the participants who maintain a high level of physical activeness acknowledged their tendency to over-exercise and tried refraining from over-exercising.

The fourth divergent phase in which the participants have an attitude of seeking improvement to reach a goal with a lot of effort is identified as *achievement striving*. Nine participants mentioned *setting their exercise-related goals*. For example, the participants quantified whether they completed the tasks and tried to carry the daily goal through. Elvin described the sequence of self-workout that he was doing every day at home. Seven participants mentioned that they *created challenges for self-improvement* when doing their physical activity. One participant started to use aqua aerobic gloves to increase resistance in the aquatic environment. 

The fifth divergent phase, *active experimentation*, can be characterized as the process of trying out a new idea or method to find out more effective ways. Nine participants mentioned that they *adopted suitable exercise or knowledge from varied experts*. For example, Karolyn said, “I do each one came from a different therapist or exercise class or something like that, so I put together exercises that I’ve learned from a lot of different people”. Eight participants described their efforts of *understanding the physiological and psychological mechanisms*. One of the participants explained how he tried to apply his understanding of motor mechanics to his golf practice. Sean stated the following:

“It works almost like a spring or a tension… when you take your golf swing, you go back here, you come through. This is you take it back here, and you caulk here. And then when you swing through you, and it gives it a lot more speed, a lot more power”.

The sixth divergent phase, *immersion*, is defined as a state of engrossment or active involvement in that activity. Ten participants mentioned their personalities in their physical activity as *focusing on what they are doing in exercise*. For example, Stanley highlighted a great deal of concentration when exercising. He said, “I’m definitely into it … I’m not interested in listening to music when I’m exercising. I have a focus here” (Stanley). Nine participants mentioned their experience when *fully immersed in the process of the activity*. The participants experienced a flow state [40,41] in which they were fully involved and enjoyed the process of physical activity losing track of time.

The seventh divergent phase, *routinization*, can be characterized as a practice of regularly performing activities that are performed as a normal part of daily life. All 15 participants mentioned that they *kept exercise in a routine*. Sherry said, “It’s a habit. It’s learning a habit and doing it. And if you can do that. You can do almost everything”. Ten participants mentioned their habits of *performing easy exercises or posture correction throughout the day*. For example, one participant mentioned that she intentionally created a challenge in her daily activity to strengthen muscles. Bonnie said, “When I get up from a chair, I don’t hold on to the side. I really get up without these [arm rest]”.

#### 3.3.2. Convergent Process

In the convergent process, the participants found commonalities based on multiple pragmatic knowledge of life to resolve issues they were confronted with. The convergent process includes the categories of (1) *self-cognizance*, (2) *reconciliation*, (3) *moderation*, (4) *adaptation*, and (5) *transcendence*. 

The first convergent phase begins with the realization of one’s own physical and mental conditions and abilities, which is identified as the category of *self-cognizance*. Ten participants emphasized self-cognizance by *analyzing self in terms of their exercise/body* as they get older. For example, Meghan stated, “Sometimes I have to realize that I have my own limitations physically. And I have to honor those or suffer the consequences. And depending on how much I’ve had to suffer the consequences and how much it hurt, I either listen or don’t”. 

Ten participants emphasized the importance of understanding their physical limitations by *being cognizant of their body’s threshold*. 

The second phase of the convergent process is identified as a *reconciliation* in which the participants find ways between seemingly opposite things in which they could be successful. When asked to describe their current physical activity and future intention, 11 participants described their intention to *maintain current exercise without challenges or a new exercise*. For instance, Elvin said, “I really haven’t thought about any new things. I’ve been doing the same exercise”. Eight participants mentioned how they *compromise between the ideal or younger and their present self*. For example, Stanley said, “Now, it’s a matter of endurance … So, I don’t work out with the intensity that I used to work at. I work with more longevity in mind”.

In the third phase of the convergent process, participants tended to make sure their activities were not too extreme, demonstrating *moderation*. Ten participants showed an unpretentious attitude toward other older people by *trying not to boast of their exercise ability or preach*. For example, Nicole said, “My friends from high school that I’m still close to, don’t have the advantages that I do. So, I don’t like to say to them, it’s almost like bragging”. Eight participants described their measured attitude when seeing age-related body changes by *giving themselves permission to not be perfect*. Tacey said, “I don’t necessarily look better than they do… I don’t think everybody needs to try to be perfect, you know, I just don’t think that’s that important”.

The fourth phase of the convergent process, which is a participant’s act of changing their personal behaviors or beliefs to make it suitable for a new situation or purpose, could be identified as an *adaptation*. This category can be divided into three subcategories of (a) *assimilation*, *accommodation*, and *integration*. *Assimilation* can be defined as a process where individuals maintain or protect a sense of self-consistency to circumvent negative feelings when facing an unpleasant situation. When asked to describe how they think about what they look like with age, six participants expressed that they managed their age-related changes in physical ability by *accentuating things they still can do*. Karolyn said, “I am trying to ignore that because if you start thinking about a problem, it’s likely to get worse”. *Accommodation* is defined as a process where individuals adjust their identity to better fit the new situation when facing an unpleasant situation. Ten participants acknowledged *human mortality and the finitude of life* that comes with age. Emilia described her modified exercise motive as she experienced her physical decline, “I don’t expect to live much longer … how much, 93? But I want it to be as healthy as possible. If I’m going to live for a long time, I want to be healthy. Yeah, I don’t want to be living like vegetating”.

*Integration* can be defined as an act or process of uniting segments to a mature and holistic state by becoming closely linked to a new and existing self. Thirteen participants responded to age-related physical changes by *living with them*. Diane said, “I just accept it. There’s no point in fighting it, you can’t do anything about it. You’ve got arthritis in your knees … just live with it and adapt”.

The final phase of the convergent process, which is the participants’ insight or way of behaving that lies beyond the practical experience of ordinary people that cannot be understood by ordinary reasoning, could be identified as *transcendence*. Eleven participants emphasized a *sense of humor* or they joked and laughed when they talked about something unpleasant but irresistible. When asked to describe the participants’ perception of their body image with age, many participants gave a witty answer. For example, Meghan said, “I wear looser tops [laughing]… Just realize it’s, it is what it is. And I can either accept it, or I can find it and be miserable. And I don’t want to do this. Life’s too short”. Six participants mentioned that there was something they did not know at a younger age but realized in later life, which can be called *wisdom-related knowledge* from their years of life experiences. For example, Elvin advised the younger generation, including me, to make a habit of exercise earlier in adulthood.

#### 3.3.3. Socioemotional Process

The categories of *social interaction* and *retrospection* occurred throughout both divergent and convergent processes as the participants’ socio-emotional aspects. The participants’ belief based on the notion that meanings are shared and constructed between people was characterized as the category of *social interaction*. Thirteen participants had people who they regularly talked with, and they *valued social interaction*. The participants’ act of recalling things past, especially in their personal experience related to physical activities and people, was characterized to be the category of *retrospection*. Thirteen participants expressed their feelings of nostalgia by *reminiscing about activities or situations*. For example, the participants reminisced about enjoyable moments of particular activities and simultaneously described the memories of their family members and friends with whom they enjoyed the activities. 

### 3.4. Age Subgroups and Underlying Health Conditions

The participants’ strategies to respond to the core category could be characterized by their age subgroup and underlying health conditions. In the following section, the categories are written in italics and the properties of the categories are written in underlined italics.

#### 3.4.1. Younger-Old Group (60s–70s Age Group)

Participants aged between 67 and 77 years were categorized into the “younger-old” group in this study based on our coding and theoretical sampling processes. The prominent categories among the younger group had the properties—“characteristic of a category” [25] (p. 101)—of (1) *extroversion*, (2) *individual orientation*, (3) *divergent thinking*, (4) *enthusiasm*, and (5) *vitality*. Table 5 and Table 6 provide the categories and properties of the age-based subgroups.

Codes related to the property of *extroversion* were identified 83 times within the younger group, whereas the frequency in the older group was only 40 times. For example, one participant mentioned that he felt full of energy in his everyday exercise and carved out time for physical activity out of a busy schedule. Stanley (76 years old) stated the following:

“Every day, every, every day, and most of the time I feel like I should be doing more. But the business has eaten up my time. We can be in the office all day. So, we’re making a concerted effort both my wife and myself making a concerted effort to try to carve out time each day for us to do that”.

Another participant mentioned that she tried to act energetically to exhibit a positive image to her grandchildren. Aritie (72 years old) said, “I don’t want to be a grandmother in a wheelchair. That’s just my motivation to be active and to be with my family, active. That’s why I do the exercise”.

Codes relating to the property of *individual orientation* appeared 81 times within the younger group whereas it occurred only 44 times in the older group. For example, the following two quotations depict participants’ individual-oriented, self-initiated physical activity: “I call, I work alone. I would like to go with somebody, but the other people, the other ladies, they have another schedule, but I like to do it alone” (72 years old, Artie); “I find myself being very comfortable being alone, dealing with the issues myself, the treadmill or the workout I don’t need somebody there to encourage me” (76 years old, Stanley).

Most of the participants in the younger group emphasized that they did not mind engaging in the social type of exercise but preferred solitary exercise.

The property of *divergent thinking* appeared as a code 39 times within the younger group, whereas the frequency in the older group was only 14 times. For example, Sean (69 years old) highlighted how he adapted new knowledge and tips for muscle-strengthening exercises from his son:

“He (my son) has now gotten me, we are working on different sections to give those muscles a chance to recuperate, where I would work on chest and arms over and over again, and what he is teaching me is that when I do that, I tear the muscle down, but I don’t give the muscles the chance to recover”.

Codes related to the property of *enthusiasm* appeared 36 times within the younger group, whereas the frequency in the older group was only 11 times. For example, one participant highlighted that she eagerly goes for a walk to relieve her stress. Artie (72 years old) said, “When I’m, when I get angry or upset or something, I go on a walk and I walk, and I walk. And after that, I feel better. They help me all the ways in, mentally, physically, emotionally”. Annika (67 years old) mentioned how she ardently engaged in her physical activity aided by an electronic fitness device. 

Codes related to the property of *vitality* were identified seven times within the younger group, while it appeared only two times in the older group. The participants in the younger group tended to do exercise that exceeds their physical limitations and recognized over-exercise. Stanley (76 years old) noted the following:

“My biggest concern is that I am energetic enough and enthusiastic enough that I find it very, very easy to overdo it. I can go too far. And I feel like I’m not doing enough again, need to do more … And my wife is constantly reminding me that I’m doing too much I’m trying to do too much. That’s the biggest issue”.

#### 3.4.2. Older-Old Group (80s Age Group)

Participants aged between 81 and 89 were categorized into the “older-old” group in this study. The prominent categories among the older group had the properties of (1) *precaution*, (2) *reliance on experts*, (3) *convergent thinking*, and (4) *resignation*. Codes related to the property of *precaution* appeared 32 times within the older group, whereas it appeared in the younger group only seven times. One participant, who had heart failure, emphasized the need for preventative behaviors in the pandemic. Emilia (83 years old) noted the following:

“I am more careful because I’m in that very dangerous age group. And I do have heart failure, so I’m aware that atrial fibrillation… I’m aware that I’m vulnerable. So, I’m very cautious. When they tell me to stay at home, I stay at home. They want us to wear the mask, I wear a mask”.

The participants in the older group highlighted the importance of fall risk prevention. Kayla (89 years old) noted, “Particularly, I go a little slower because I have to be careful that I don’t slip and I don’t fall”. 

Codes related to the property of *reliance on experts* occurred 27 times within the older group, and but only six times in the younger group. When asked to describe the participants’ experience of when they were able to improve exercise skills and participate in the activity over a long period of time, most of the participants in the older group referred to taking an exercise class. For example, the participants demonstrated a tendency to rely on the instructor, “Just with the instructor. She demonstrates it and tells us what she wants us to do” (81 years old, Bonnie); “I do whatever they [instructors] asked me to do. And whenever it gets better weather and they allow us, we will go to the pool, not that I swim. But I will do the exercise” (89 years old, Kayla).

Codes related to the property of *convergent thinking* appeared 25 times within the older group, whereas it occurred only three times in the younger group. For example, the participants in the older group mentioned that they came to acknowledge their own mortality, which was based on their accumulative experiences and knowledge, “I realize when I play golf with my peer group… We talked about how many of our teammates for the football or golf, they’re passed away, and that they were much better athletes than I was” (81 years old, Elvin). Nicole (84 years old) described how she eased unpleasant feelings about her own age-related physical decline and became used to the physical changes by self-evaluation. 

Codes related to the property of *resignation* were identified 23 times within the older group, whereas it occurred only four times in the younger group. When asked to describe their experience of new physical activities in the last few years and their intention to pursue new physical activities, all participants of the older group agreed that they would maintain their same current physical activity without adding challenges or new exercises. For example, the participants showed their intention not to explore new exercises, “No, I don’t do that very much. I kind of stick to the same things” (85 years old, Sherry); “I don’t think it’s necessary [to do new exercise]” (83 years old, Karolyn); “I haven’t really added. I wouldn’t” (84 years old, Nicole); “I tend to be more repetitive. I stick with what I know” (83 years old, Emilia).

#### 3.4.3. Without Underlying Health Conditions

The prominent categories among the participants without underlying health conditions had the properties of (1) *enthusiasm*, (2) *divergent thinking*, and (3) *extroversion*. Table 7 and Table 8 provides the categories and properties of the subgroups depending on the participants’ underlying conditions.

Codes related to the property of *enthusiasm* appeared 68 times among the participants without underlying conditions, whereas it was represented by 43 codes among the participants with underlying conditions. For example, Clifford (84 years old) stated the following:

“Just to be active and play regularly … And I go to tournaments every once in a while. I play two, three, couple, three tournaments a year that come up, where you’re playing against people from other states or other towns. Some of those are goals to keep yourself ready, capable to participate in those events”.

Elvin (81 years old) also mentioned how he set the specific exercise goal and tried to reach that goal:

“Now it’s got to be sort of a challenge to be able to hit that 33 separate times in the morning. Someday will be a day in the future, whether that’s going to be tomorrow, or when it’s going to be a year or two years from now three years from now, I will be able to hit that”.

Codes related to the property of *divergent thinking* appeared 33 times amongst participants without underlying conditions, and 20 times for the participants who had underlying conditions. Annika (67 years old), who had no underlying conditions, said, “Just try to do as much as you can and still feel good about what you’re doing. Because you’re still getting those endorphins, you’re still feeling good about what it is that you’re doing because you’re working out”. Sean (69 years old) highlighted how he has engaged in his exercise by actively exploring the physiological mechanism:

“Also doing things and then not keeping them up is also painful. You go through the process of exercising and go through that soreness period, and then you stop. And then you have to start all over again and go through that whole process again”.

Codes related to the property of *extroversion* appeared 27 times among the participants without underlying conditions, but appeared only three times for participants with underlying conditions. For example, Sean (69 years old) mentioned how he intentionally set aside time for his practice out of his working schedule. The other participant actively adopted new technology for exercise, thereby enhancing his exercise effect. Clifford (84 years old) said, “It’s a golf GPS… If you are going to shoot par… you want to know how far it is. So which club you can use. So, it’s essential to playing the game very competitively”.

#### 3.4.4. With Underlying Health Conditions

The prominent categories among the participants with underlying health conditions had the properties of (1) *convergent thinking*, (2) *communion*, (3) *reliance on experts*, (4) *resignation*, and (5) *wistfulness*.

Codes related to the property of *convergent thinking* appeared 89 times for participants who have underlying conditions, but it appeared only five times for participants without underlying conditions. The participants who have one or multiple underlying health conditions tried not to be critical of their age-related physical changes and accepted their current physical conditions by continuing what they can do, “I try not to be critical. Just try to take care of it” (Karolyn, 83 years old); “I do the best I can with the exercises. So far so good” (Bonnie, 81 years old).

The other participant tried to think positively when it comes to their health and physical activity. For example, when Emilia (83 years old) delineated her story of how she was cured from cancer, she positively attributed her illness and treatment.

Codes that were related to the property of *communion* were identified 49 times among the participants with underlying conditions, but only five times for participants without underlying conditions. For example, the participants with underlying health conditions emphasized their empathic feelings with the other peers in the exercise class or sports club and valued the social effect of exercise. Tacey (88 years old) said, “I just enjoy being with the people that I’m exercising with because we still need to socialize as we get older”. They also emphasized the importance of building camaraderie with peers who have physical and psychological commonalities. Nicole (84 years old) said, “We’re all encouraging and we enjoy each other’s company”.

Codes related to the property of *reliance on experts* appeared 33 times among the participants with underlying conditions, whereas no codes were identified for the participants without underlying conditions. When asked to describe their experience of learning new skills or knowledge about the physical activity that they engaged in, 10 participants with underlying conditions tended to rely on an instructor-led class. For example, Artie (72 years old) considered the fitness instructor was the primary source of getting knowledge in terms of exercise. Kayla (89 years old) also mentioned her sole reliance on the fitness class without any self-initiation regarding physical activity.

Codes that were related to the property of *resignation* appeared 29 times among the participants with underlying conditions, whereas the frequency for the participants without underlying conditions was only five times. When asked to describe their goals of physical activity, five participants with underlying conditions mentioned maintaining physical functioning or independence, rather than the advancement of physical ability. They expressed their resigned attitude in exercise, “To just do it, so that I’m staying. I don’t know that I have any particular goal, I’m not trying to get better at it. I’m just trying to keep myself in good shape” (Karolyn, 83 years old); “I’m doing exercise, so it could be much worse. It is, it is not painful, but it’s not completely fine, but I’m doing okay. That is the only thing I could say that bothers me” (Artie, 72 years old).

Codes related to the property of *wistfulness* appeared 22 times among the participants with underlying conditions, and four times for participants without underlying conditions. The participants with underlying conditions expressed their feelings of longing for varied physical activities they had actively engaged in in the past. Artie (72 years old) who has a back issue said, “I must say, at my age… Yes, sometimes it is hard. It’s hard for me when I cannot do things like before”. While the participants expressed their feelings of missing the past when they more freely performed various movements, they also acknowledged their physical changes. Meghan (77 years old) stated, “We did some mountain climbing and things like that. I can’t do that kind of thing anymore. But it was fun! Yeah, we did it … [I have] been there done that. I don’t need to do it again. I’m not gonna go climbing a mountain anymore”.

### 3.5. Conceptualization of Physical Identity Shift in Later Life

**Figure 1:**
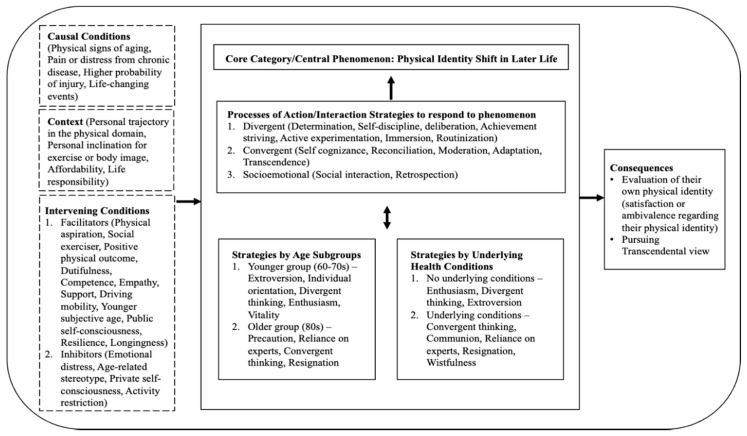
Model of physical identity development in later life.

All the categories described in the open, axial, and selective codes contributed to the core category, which is depicted as a diagram. We used diagramming as a tool for integrating the categories and presenting the final theory [24]. The schematic diagram represents data analyzed from multiple rounds of interviews (see Figure 1). Each of the rectangular boxes with a solid line represents a part of the paradigm model (i.e., phenomenon, action/interactional strategies, and consequences) that played a major role in explaining the physical identity development process model. Three rectangular boxes with a dotted line represent conditions that led to the phenomenon (i.e., causal condition), facilitated or constrained the strategies (i.e., intervening conditions), and the participants’ personal context that pertained to a phenomenon (i.e., context).

The participants’ physical identity shift (i.e., phenomenon) to better adjust to their aging bodies and physical changes may be caused by the physical signs of aging, pain or distress from chronic disease, a higher probability of injury, and life-changing events (i.e., causal conditions). The contextual conditions within the process of physical identity shift were each individual’s different personal trajectory in the physical domain, personal inclination for exercise or body image, affordability, and life responsibility (i.e., context). The conditions of physical aspiration, social exerciser, positive physical outcome, dutifulness, competence, empathy, support, driving mobility, younger subjective age, public self-consciousness, resilience, and longingness (i.e., intervening conditions—facilitators) acted to promote the participants’ strategies for how they processed their physical identity development and learned to adjust. In contrast, the conditions of emotional distress, age-related stereotype, private self-consciousness, activity restriction (i.e., intervening conditions—inhibitors) hindered the participants from effectively processing their physical identity development. The participants managed the phenomenon through determination, self-discipline, deliberation, achievement striving, active experimentation, immersion, and routinization (divergent process); self-cognizance, reconciliation, moderation, adaptation, and transcendence (convergent process); and social interaction and retrospection. Consequently, they may end up reaching states of satisfaction with their own physical identity or ambivalent emotions regarding their physical identity and pursuing a more transcendental view.

## 4. Discussion

### 4.1. Physical Activities and Perceptions of Aging

Our findings addressed that a convergent phase started when the participants began to realize their age-related changes in physical or mental conditions and abilities. While engaging in physical activities, they were attentive to bodily sensations and appropriated multiple pragmatic forms of knowledge from their life experiences to relieve the discrepant feelings originating in their aging bodies. This finding is supported by the prior studies regarding the relationships between involvement in physical activity, bodily awareness, and perception of aging [42,43]. Fougner et al. [43] found that older-women exercisers showed body awareness and eagerness to understand their bodily sensations and signs of aging during exercise participation. Similar to Erden and Güner’s [42] study regarding the effects of body awareness on the emotional condition, pain, and quality of life among older adults, in this study, the participants who demonstrated high sensitivity to detecting the internal bodily signal expressed confidence to respond to age-related physical changes.

This finding can also be explained to some extent by an active aging model [44] that suggests, “a general lifestyle strategy for preservation of physical and mental health as people age” [45] (p. 124). Our findings corroborated the findings of a great deal of the previous work in older adults’ involvement in active aging [46,47]. Similar to Mendoza-Ruvalcaba and Arias-Merino’s [47] study, which found that the involvement in an active aging promotion program brought about older adults’ improved dimensions of active aging (i.e., physical activity, nutrition, cognitive function, and quality of life), the participants in this study mentioned that their regular participation in a senior fitness program stimulated their optimistic view on the aging process.

The findings also challenge the rhetoric in the broader context of aging in a society that suggests exercise is a means to fight against biological aging. Consistent with the literature arguing with the notion of the anti-aging purpose of the exercise [48,49], the participants in this study did not regard aging as an object that they can defeat, but rather as being actively involved in the process of compromising between ideal physical abilities or body image and reality as they get older. In this regard, the finding paralleled Tulle’s [48] work that reconstructed the notions of the aging body in the modern society where people uncritically consider exercise as a measure of anti-aging. The participants in this study constantly customized a younger ideal body image to fit their present self-body image, in which they faced physical decline or pain while exercising, and thus they did not express the sense that they were in a battle against the natural aging process.

### 4.2. Physical Identity Development through Exercise Engagement

Our findings addressed the dichotomous processes of physical identity development among older adults. These divergent and convergent modes of coping strategies correspond to the notions of exploration and commitment respectively in traditional identity development theories [50]. Identity development can occur as the dynamics between exploration and commitment in which individuals “search for new information relevant to forming an identity” (i.e., exploration or divergent); “sort through of information and select among alternatives to arrive at a coherent set of goals and values” (i.e., commitment or convergent) [40] (p. 219). In this way, identity development works best when individuals operate in both divergent and convergent processes [41,51].

Our findings, particularly the category of adaptation within the convergent process, addressed the process of how older adults cope with age-related physical changes. This finding regarding the category of adaptation can be explained by Baltes’s [52] theory of Selection, Optimization, and Compensation (SOC). This theory accounts for the “allocation of resources into the three major functions of ontogenetic development: growth, maintenance (resilience), and the regulation or management of loss” (p. 377). The participants in our study demonstrated their own adaptive process for countering loss and decline across their lifespan. In this regard, our finding paralleled one of the key concepts from Baltes’s [52] SOC theory that the primary resource allocation tends to be toward compensation in old age. The major subcategories of adapting age-related physical changes among older adults were the assimilation and accommodation of aging effects. Assimilation can account for a way that older adults maintain their existing identity by adjusting external factors with increasing age. For example, participants acquired new exercise skills and formed exercise-related habits as ways to resolve negative physical effects or feelings from age-related physical decline or aging body image. The other major process of accommodation explains ways older adults reconstruct their existing identity by adjusting internal factors. For instance, the older adults actively participated in a reflection in terms of their exercise practice, became more aware of bodily sensations during activities, or brought nostalgia about the activities that they enjoyed in youth.

The finding regarding the assimilation and accommodation processes is explained by identity process theory that explains the process of how people negotiate discrepant feelings when facing unwanted new experiences [53,54]. Such emotional distress from confronting new experiences that conflict with their existing identity is called “identity threat” [53,55]. In this study, older adults’ perception of aging bodies promoted their physical identity threat that they had not worried about when they were younger. Some older adults in this study used assimilation as their primary coping mechanism when confronting age-related physical changes, and others used accommodation. However, these two modes of the process were not seen as mutually exclusive, rather sometimes operating simultaneously. Consistent with the prior identity threat studies [56,57], the findings emphasized that assimilation helped older adults circumvent negative feelings from aging effects by enhancing exercise skills or transforming their exercise routine. Likewise, the studies employing identity process theory [58,59], this study also addressed accommodation as a primary coping mechanism for older adults when dealing with age-related physical changes. The participants had revised their values and priorities in a way that they reevaluated their physical limitations with age, constructed new meanings in health, and thus transformed their physical identity.

Findings from this study have implications for identity process theory and aging studies. The existing identity process theory accounted for the process of identity shift to be assimilation, accommodation, and a plateau-like stage of integrated balance [53,54]. We could echo the major concepts of existing identity process theory and further elaborate a contextual description of identity process theory. In this study, certain states that older adults reached through the process of identity development were satisfaction with their own physical identity or transcendental experience by acknowledging the finitude of the human body. These states aligned with the existing concept of assimilation and accommodation in the identity process theory and reinforced the mechanism of how individuals negotiate the experience of aging and existing identity [53,54]. Further, the older adults’ strategies of managing age-related physical changes in this study emphasized the iterative characteristic of physical identity development. Thus, older adults may keep engaging in their physical identity shift process throughout their life course as an ongoing process, rather than in a teleological, linear way. This can inform people’s understanding and help them acknowledge that constant maintenance of one’s physical self may be required indefinitely.

### 4.3. Various Age Subgroups and Underlying Health Conditions

The next emerging categories showed older adults’ coping strategies for dealing with the physical changes that come with age, as varied by their age grouping and the severity of underlying health conditions. This finding of different characteristics within an older population can be explained, to some extent, by previous studies’ subgrouping older people into young-old (65 to 74 years), middle-old (75 to 84 years), and old-old (85 years and older) groups such as different psychological aspects [60], biological differences [61], different health behaviors [62], and different learning styles [63]. However, older adults’ differences in coping strategies for how to respond to age-related physical changes have not been explicitly studied. Our research addressed this gap by identifying specific characteristics that younger-old and older-old adults demonstrated. For example, the coping strategy style of the younger-old group (e.g., individual orientation, divergent thinking, or enthusiasm) was somewhat contrary to the characteristics of the older-old group (e.g., reliance on experts, convergent thinking, precaution, or resignation).

This finding related to older adults’ severity of underlying conditions somewhat contributed to demonstrating the contrasting characteristics among the participants. This discovery of different effects related to one’s underlying health conditions on their perceptions of aging can be explained by previous studies regarding the relationship between fatigue qualities and underlying conditions among older adults [64], and the effect of underlying health conditions on older adults’ new disability [65]. However, we could not find studies regarding various coping strategies based on older adults’ severity of underlying conditions. In this way, the present findings can contribute to explaining the various coping strategies older adults employ, such as those without underlying conditions (e.g., enthusiasm, divergent thinking, and extroversion) and those with underlying conditions (e.g., convergent thinking, communion, reliance on experts, resignation, and wistfulness) as distinguishable characteristics.

The present findings also highlight older adults’ heterogeneity. In much previous aging research, older adults were seen as homogenous in terms of age, race, class, ethnicity, and able-bodiedness [66]. This study emphasizes the characteristics and coping strategies as differentiated between older adults’ age subgroups and underlying health conditions. For example, participants in the younger-old group and those without underlying health conditions showed the characteristics of extroversion and divergent thinking, while participants in the older-old group and those who had underlying health conditions showed resignation, convergent thinking, and reliance on experts. In this regard, this finding emphasizes that human variability remains throughout the lifespan.

This finding has practical implications for instructors, program coordinators, and directors of senior institutes. Our findings regarding different characteristics based on younger- and older-old adults can inform practitioners in senior centers to better understand the significant variability among the senior clients. An older population can be characterized as heterogeneous because of their variability in the aging experience, which also implies varied directions of development across individuals or groups [67,68]. Thus, older adults showed different interests in terms of the purpose and types of exercise and perceptions of self-body as they get older. A better understanding of age-specific characteristics and interests can help practitioners to identify appropriate educational programs and physical activities that improve older adults’ physical and psychological health. In this way, when developing senior programs and activities, older adult educators or instructors should consider various interests and thus they can provide a wider variety of age-specific activities. For example, senior activity programs should also reflect the senior members’ different psychological aspects [60], biological differences [61], different health behaviors [62], various cultural beliefs about aging, and different learning styles [63] based on age subgroups.

## 5. Limitations and Future Directions

Participants in this study were active or highly active in terms of levels of physical activity and that they are regularly engaged in a physically active lifestyle. However, 28% of adults aged 50 and older in the United States are still categorized as physically inactive [9,10]. Therefore, the findings may be limited to such a particular subgroup of the older adult population and cannot be generalized to other older populations with different levels of physical activeness. Future research may investigate the experiences of older adults who are less physically active, or inactive. Moreover, examining the varied exercise status among older adult subgroups including non-exerciser, sociable non-exerciser, active living, solitary exerciser, social exerciser, and extreme exerciser may also illuminate older adults’ experiences and perceptions of health and participation in physical activity that are different from what we found in this study.

## 6. Conclusions

Older adults become more attentive to the condition of their health and physical functions than they were in their younger years, considering the major perspectives on adult development of biological, psychological, and sociocultural aspects. However, developing one’s physical sense of self in later life may be a challenging endeavor that requires constant evaluation and adaptation of one’s own physical changes and identity. The primary findings of the analysis were a final model depicting (a) divergent and convergent modes of strategies and socioemotional aspects of physical identity development in later life and (b) different strategies employed between younger-old versus older-old age groups and between participants who have underlying health conditions and those who do not. Future research may take different levels of physical activeness and various demographics into account.

## Figures and Tables

**Table 1:** Theoretical sampling.

	60s–70s Age Group	80s Age Group
With Underlying Health Conditions	72 years, pinched nerve in the back 76 years, heart failure, pacemaker 77 years, chronic back pain	81 years, arthritis, osteoporosis, heart issue, replacement knee 83 years, scoliosis 83 years, vision impairment, heart failure 84 years, bad knee, feet issues, back pain 85 years, back issue 88 years, severe back problems, nerve pain 89 years, heart issue, hearing issue
Without Underlying Health Conditions	67 years 69 years	Analytical need for participants who were in their 80s without underlying conditions

**Table 2:** Utilizing the paradigm model in axial coding.

Elements of the Paradigm Model	Categories
Phenomenon	Physical identity shift in later life
Causal conditions	Physical signs of aging, pain or distress from chronic disease, a higher probability of injury, life-changing events
Context	Personal trajectory in the physical domain, personal inclination for exercise or body image, affordability, life responsibility
Intervening conditions (inhibitor)	Emotional distress, age-related stereotype, private self-consciousness, activity restriction
Intervening conditions (facilitator)	Physical aspiration, social exerciser, positive physical outcome, dutifulness, competence, empathy, supports, driving mobility, younger subjective age, public self-consciousness, resilience, longingness
Action/Interactional Strategies	Determination, self-discipline, deliberation, achievement striving, active experimentation, immersion, routinization, self-cognizance, reconciliation, moderation, adaptation, transcendence, social interaction, retrospection
Consequences	Evaluation of their own physical identity (satisfaction or ambivalence regarding their physical identity), transcendental views

**Table 3:** Participants’ demographics.

Pseudonym	Gender	Age	Main Activity	Levels of Physical Activity	Exercise Status/Preference
Sherry	Female	85	Senior exercise program, gym, walking, water aerobics	Active	Active living, social exerciser
Karolyn	Female	83	Self-workout at home, Senior exercise program, walking	Active	Active living, social exerciser
Meghan	Female	77	Senior exercise program, self-workout at home, walking	Active	Active living, social exerciser
Bonnie	Female	81	Senior exercise program, walking, water aerobics	Highly active	Active living, social exerciser
Sean	Male	69	Golf, gym	Highly active	Solitary exerciser (but engaged in social exercise groups)
Nicole	Female	84	Senior exercise program, water aerobics, walking	Active	Active living, social exerciser
Artie	Female	72	Senior exercise program, walking, swimming	Active	Active living, solitary exercise
Tacey	Female	88	Senior exercise program, walking, water aerobics	Active	Active living, social exerciser
Kayla	Female	89	Senior exercise program, walking	Active	Active living, social exerciser
Emilia	Female	83	Senior exercise program, gym	Active	Social exerciser
Stanley	Male	76	Golf, walking, self-workout at home	Highly active	Active living, solitary exerciser
Annika	Female	67	Walking, self-workout at home	Highly active	Active living, solitary exerciser
Clifford	Male	84	Golf, gym, walking	Highly active	Social exerciser
Diane	Female	74	Walking, golf, cycling	Active	Solitary exerciser
Elvin	Male	81	Self-workout at home, golf, walking	Highly active	Active living, social exerciser

**Table 4:** Three traits of the process in action/interactional strategies.

Trait of Process	Categories	Definition
Divergent	Determination	Strong decision to continue trying to do something despite the difficulty
	Self-discipline	Ability to begin the exercise-related tasks and carry them through to the goal achievement
	Deliberation	Careful consideration before taking action or situations that occur
	Achievement striving	Attitude of seeking improvement to reach a goal with a lot of effort
	Active experimentation	Process of trying out a new idea or method to find out more effective ways
	Immersion	Engrossment or active involvement in that activity
	Routinization	Practice of regularly performing activities that are performed as a normal part of daily life
Convergent	Self-cognizance	Realization of one’s own physical and mental conditions and abilities
	Reconciliation	Process of finding a way between seemingly opposite things in which both can be successful
	Moderation	Acting in a way that is not extreme
	Adaptation	Act of changing personal behaviors or beliefs to adapt to a new situation or purpose
	Transcendence	Insight or way of behaving that lie beyond the practical experience of ordinary people, and cannot be understood by ordinary reasoning
Socioemotional (throughout the process)	Social interaction	Belief based on the notion that meanings are shared and constructed between people
	Retrospection	Act of recalling things in the past, especially in one’s personal experience about physical activities

**Table 5:** Younger-old group (60s–70s).

Property	Definition of Property
Extroversion	A tendency to be outgoing, talkative, and exhibit energetic behavior
Individual orientation	A tendency to emphasize one’s personal goals and achievement
Divergent thinking	A thought process distinguished by exploring multiple ideas to generate new possible solutions to problems
Enthusiasm	Great eagerness to be involved in a particular activity that one enjoys or thinks is important
Vitality	A state of being strong, active, and enthusiastic

**Table 6:** Older-old group (80s).

Property	Definition of Property
Precaution	An action that is intended to prevent dangerous or unpleasant situations from happening
Reliance on experts	A tendency to do things and make decisions with advice from experts in terms of physical activity and health
Convergent thinking	A thought process of integration based on existing knowledge and experiences to come up with a single, well-established answer
Resignation	Acceptance with reluctant feelings, but knowing that something cannot be changed

**Table 7:** Without underlying health conditions.

Property	Definition of Property
Enthusiasm	Great eagerness to be involved in a particular activity that one enjoys or thinks is important
Divergent thinking	A thought process distinguished by exploring multiple ideas to generate new possible solutions to problems
Extroversion	A tendency to be outgoing, talkative, and do energetic behavior

**Table 8:** With underlying health conditions.

Property	Definitions of Property
Convergent thinking	A thought process of integration of existing knowledge and experiences to come up with a single, well-established answer
Communion	A state of a sense by which one shares or exchanges intimate thoughts and feelings with others
Reliance on experts	A tendency to do things and make decisions as advised by experts
Resignation	Acceptance with reluctant feelings, but knowing that something cannot be changed
Wistfulness	A state of a sense of missing something that one can never regain

## Data Availability

Data sharing is not applicable to this article.
